# Copper death combination therapy: the innovative frontier and challenges in prostate cancer treatment

**DOI:** 10.1080/15384047.2025.2532224

**Published:** 2025-07-15

**Authors:** Jia Wei He, Pei Zhen Li, Zi Xuan Huang

**Affiliations:** a Department of Urology, Dongguan Songshan Lake Central Hospital, Dongguan, China; b Department of Orthopedic Surgery, Dongguan Eighth People’s Hospital, Dongguan, China

**Keywords:** Prostate cancer, copper metabolism, androgen receptor, therapeutic strategies, drug resistance

## Abstract

Prostate cancer (PCA) remains a significant health challenge, necessitating the exploration of novel therapeutic strategies to enhance patient outcomes. Recent research has identified cuproptosis, a copper-dependent programmed cell death mechanism, as a promising target in PCA treatment. Elevated copper levels have been associated with tumor progression and therapeutic resistance, highlighting the need for innovative approaches. This review synthesizes current findings on the role of copper and cuproptosis in PCA, focusing on the mechanisms underlying cuproptosis, the identification of key biomarkers, and the therapeutic potential of copper complexes and ionophores. The integration of cuproptosis-related biomarkers into clinical practice may facilitate personalized treatment strategies, while ongoing research into copper-based therapies holds promise for overcoming limitations of traditional chemotherapy. Future directions should emphasize elucidating the molecular mechanisms of cuproptosis and optimizing therapeutic applications to improve patient outcomes in PCA.

## 1. Introduction

PCA is one of the most prevalent malignancies affecting men worldwide, representing a significant public health concern due to its high incidence and mortality rates. It is the second leading cause of cancer-related deaths among men, with a notable increase in reported cases in recent years.^1^ The complexity of PCA, particularly in its advanced stages, necessitates the exploration of novel therapeutic approaches to improve patient outcomes and address the challenges posed by treatment resistance. The significance of innovative treatment strategies is underscored by the limitations of current therapies, which often exhibit poor efficacy and severe side effects.^2^ Traditional chemotherapeutic regimens are frequently hindered by the development of drug resistance, especially in metastatic cases where tumors become refractory to established treatments.^3^ This situation highlights the urgent need for alternative therapeutic modalities that can effectively target the unique biological characteristics of PCA.

Recent research has identified copper and its role in tumor progression as a promising area of investigation. Elevated levels of copper have been observed in various cancers, including PCA, where it is implicated in tumor growth and metastasis.^4^ In a prospective study, Lubiński et al.^5^ measured the associations between serum copper levels and prognosis in PCA patients, finding that higher copper levels were associated with increased mortality, thus reinforcing the significance of copper in cancer biology.^5^ The concept of cuproptosis, a copper-dependent programmed cell death mechanism, has emerged as a critical factor in PCA development and therapeutic resistance (Cheng et al.^3^). This process involves the direct binding of copper ions to specific components of the tricarboxylic acid cycle, leading to proteotoxic stress and cell death, thereby presenting a potential target for therapeutic intervention (Wu et al.^2^). Furthermore, the development of copper-containing nanomedicines has shown promise in overcoming the limitations associated with traditional chemotherapy (Wu et al.^2^). These novel therapeutic agents leverage the unique properties of copper to induce selective cytotoxicity in cancer cells while minimizing damage to normal tissues. This approach aligns with the growing emphasis on personalized medicine, where treatment strategies are tailored to the individual characteristics of both the tumor and the patient.

In summary, the exploration of copper and cuproptosis in the context of PCA represents a significant advancement in cancer research. The integration of these novel therapeutic strategies may pave the way for more effective treatment options, ultimately improving survival rates and quality of life for patients diagnosed with prostate cancer. Additionally, the potential of radiopharmaceutical therapy, which delivers cytotoxic radiation doses precisely to malignant tumors, has shown notable success in metastatic, castration-resistant PCA, further highlighting the need for diverse treatment approaches.^6^

## 2. Probing cuproptosis for prostate cancer therapy

Copper metabolism and its implications in cancer therapy, particularly prostate cancer, have garnered significant attention in recent years. A novel form of programmed cell death known as cuproptosis has emerged as a critical area of study, highlighting the intricate relationship between copper ions and cancer progression.Cuproptosis is characterized by the direct binding of copper ions to lipoylated components of the tricarboxylic acid cycle, leading to proteotoxic stress and ultimately cell death. This mechanism is particularly relevant in the context of prostate cancer, where copper homeostasis plays a crucial role in tumor development and therapeutic sensitivity (Wu et al.^2^). Elevated levels of copper have been observed in various cancers, including prostate cancer, suggesting that copper may act as a co-factor in tumorigenesis and angiogenesis.^7^ Recent studies have explored the potential of cuproptosis in overcoming chemotherapeutic resistance in prostate cancer. For instance, Wen et al.^8^ demonstrated that cuproptosis can enhance the sensitivity of prostate cancer cells to docetaxel, a first-line chemotherapeutic agent.^8^ Their findings indicated that the cuproptosis-regulated DLAT/mTOR pathway inhibits autophagy and promotes cell cycle retention in the G2/M phase, thereby improving chemosensitivity.

The role of long non-coding RNAs (lncRNAs) related to cuproptosis has been investigated as potential prognostic markers in prostate cancer. Cheng et al.^9^ developed a cuproptosis-related lncRNA signature that has shown promise in predicting patient outcomes.^9^ This signature is closely associated with various clinicopathological traits, including age, T stage, N stage, and Gleason score, suggesting that cuproptosis-related lncRNAs could serve as valuable biomarkers in clinical settings. Additionally, Zhong et al.^10^ emphasized the necessity for further research into the association between cuproptosis-related lncRNAs and prostate cancer prognosis, highlighting their regulatory role in mRNA expression and cancer progression.^10^ The therapeutic implications of copper complexes in prostate cancer treatment have also been underscored. Bontempo et al.^11^ reported that a copper(II) complex exhibited selective cytotoxicity toward prostate cancer cell lines, significantly reducing proliferation markers.^11^ This finding suggests that copper-based therapies may provide a novel approach to treating advanced prostate cancer, potentially overcoming the limitations of traditional chemotherapy. Furthermore, Alhasawi et al.^12^ demonstrated that curcuminoids, polyphenolic compounds found in turmeric, can inhibit cell proliferation and induce apoptosis-like cell death in prostate cancer cell lines.^12^ Their study indicated that copper chelation and reactive oxygen species scavengers significantly influenced this reaction, suggesting a copper-mediated mechanism in the anticancer effects of curcuminoids.

In summary, the exploration of copper metabolism and cuproptosis presents a promising frontier in prostate cancer therapy. Understanding the mechanisms underlying cuproptosis and its relationship with lncRNAs and copper complexes may pave the way for innovative treatment strategies aimed at improving patient outcomes.

### 2.1. Mechanisms of cuproptosis in prostate cancer

**Figure 1.**
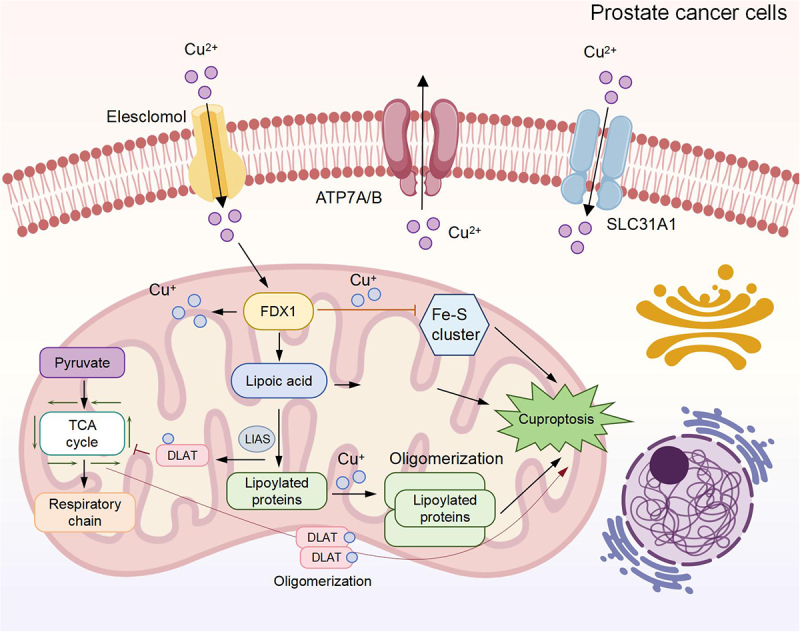
Schematic diagram of the molecular mechanism of cuproptosis in prostate cancer cells.

Cuproptosis, a newly identified form of programmed cell death induced by copper ions, has emerged as a significant mechanism influencing the progression and treatment resistance of PCA (Figure 1). Recent studies have elucidated various genetic and molecular pathways through which cuproptosis operates in PCA. For instance, Wen et al.^8^ demonstrated that cuproptosis is linked to the chemotherapeutic resistance of PCA to docetaxel, revealing that the DLAT/mTOR pathway plays a crucial role in this process. Their findings indicated that cuproptosis can inhibit autophagy and promote cell cycle retention in the G2/M phase, thereby enhancing chemosensitivity. Cuproptosis is triggered when copper ions (Cu^2 +^) bind to lipoylated enzymes in the TCA cycle, such as dihydrolipoamide acetyltransferase (DLAT). This binding disrupts iron-sulfur (Fe-S) clusters in proteins like ferredoxin 1 (FDX1), causing mitochondrial dysfunction and the release of reactive oxygen species (ROS). ROS subsequently induce proteotoxic stress through the aggregation of misfolded proteins, leading to cell death.

Furthermore, Cheng et al.^9^ explored the prognostic implications of cuproptosis-related genes (CRGs) in PCA. They identified a predictive model based on five CRGs that significantly correlated with patient outcomes and responses to immune checkpoint inhibitors. This model underscores the importance of cuproptosis in modulating the tumor immune microenvironment and influencing therapeutic responses. Additionally, Jin et al.^13^ conducted a multi-omics analysis that highlighted the alterations in cuproptosis-related genes and their prognostic significance in PCA.^13^ Their research revealed that these genes are involved in critical pathways, such as the E2F and G2M targets, which are essential for cell cycle regulation and tumor progression. Moreover, Ding et al.^14^ identified the role of amine oxidase copper-containing 1 (AOC1) as a tumor suppressor in PCA, suggesting that AOC1‘s regulation by the transcription factor SOX15 may enhance anticancer effects through mechanisms involving reactive oxygen species and ferroptosis.^14^ This comprehensive understanding of cuproptosis mechanisms provides a foundation for developing targeted therapies aimed at exploiting copper homeostasis in PCA.

A study conducted by Bontempo et al.^11^ evaluated the cellular effects of a copper(II) complex on PCA cell lines, revealing its selective cytotoxicity toward LNCaP cells while sparing normal prostate cells. This finding suggests that copper complexes may represent a promising class of metal-based drugs for the treatment of malignant neoplasms, thereby further supporting the role of copper in cancer biology. Additionally, Yu et al.^15^ investigated the prognostic value of cuproptosis-related long non-coding RNAs (lncRNAs) in PCA, developing a predictive signature capable of forecasting biochemical recurrence in patients.^15^ This underscores the potential of cuproptosis-related biomarkers in clinical applications. Furthermore, a novel family of copper(I) complexes has been synthesized and characterized, exhibiting greater cytotoxicity toward prostate cancer cells compared to normal prostate cells. Among these, the compound [Cu(dppe)(2-ap)][BF4] demonstrated marked cellular internalization and the generation of intracellular reactive oxygen species, activating cell death mechanisms through both apoptosis and necrosis. Notably, this compound’s cytotoxic activity was found to be 70-fold greater for LNCaP cells than for normal prostate cells, indicating its potential for further investigation in prostate cancer models.^16^

### 2.2. Copper-AR signaling crosstalk in prostate cancer

Copper metabolism intersects with androgen receptor (AR) signaling to drive prostate cancer progression. Androgens induce the expression of copper transporter 1 (CTR1, encoded by SLC31A1), thereby promoting cellular copper uptake and subsequent activation of AR-mediated transcription.^1^ Conversely, copper ions enhance AR nuclear translocation and DNA binding affinity, amplifying the expression of AR target genes, such as KLK3, which are involved in tumor proliferation.^2^ This bidirectional regulation forms a positive feedback loop: AR signaling upregulates copper import, while copper reinforces AR activity. In castration-resistant prostate cancer (CRPC), copper may stabilize AR splice variants, such as AR-V7, that lack the ligand-binding domain, enabling androgen-independent AR activation.^3^ For instance, Wen et al.^8^ demonstrated that the inhibition of cuproptosis via the DLAT/mTOR pathway reduces AR-V7 expression, thereby sensitizing CRPC cells to docetaxel.^8^ Furthermore, copper-mediated proteotoxic stress may disrupt AR co-regulator interactions, further modulating transcriptional output.^4^ These findings highlight the AR-copper axis as a potential therapeutic target to overcome endocrine resistance (Table 1). Combining androgen deprivation therapy (ADT) with cuproptosis inducers may effectively counteract resistance. Preclinical data indicate that the combination of enzalutamide (an AR inhibitor) with copper ionophores reduces tumor growth by specifically targeting AR-V7-positive cells.^8,18^ This approach leverages the AR-copper crosstalk to enhance therapeutic efficacy, warranting further clinical exploration.

**Table 1.** Comparative analysis of cuproptosis research in prostate cancer and breast cancer.

Comparison Dimension	Prostate Cancer	Breast Cancer
Core Mechanisms	- Copper ions enter cells via SLC31A1 and bind to lipoylated proteins (e.g., DLAT) in the TCA cycle, leading to Fe-S cluster degradation and mitochondrial stress - Androgen receptor (AR) signaling may regulate copper transporter (e.g., CTR1) expression, forming a metabolic-signaling crosstalk	- Copper ions induce aggregation of lipoylated proteins, activating FDX1-mediated mitochondrial damage - Estrogen receptor (ER) signaling is closely associated with copper metabolism, potentially influencing copper homeostasis by regulating SLC31A1
Key Molecules/Pathways	- Key genes: DLAT, mTOR, SLC31A1 - Related lncRNAs: LIPE-AS1, STPG3-AS1 (Cheng et al.^3^) - Crosstalk with AR pathway: Copper enhances AR nuclear translocation and AR-V7 stability	- Key genes: FDX1, DLAT - Related proteins: CTR1 (copper uptake), MT (copper binding) - Association with ER/HER2 pathways: Copper may affect ER activity via PI3K/AKT
Therapeutic Implications	- Enhanced chemosensitivity: Cuproptosis inducers (e.g., elesclomol) potentiate docetaxel-mediated killing of prostate cancer cells (Wen et al.^8^) - Overcoming resistance: Copper ionophores (e.g., Cu(DDC)₂) are effective against drug-resistant CRPC cells (Chen et al.^17^)	- Synergy with targeted therapy: Cuproptosis combined with paclitaxel enhances apoptosis in breast cancer cells (Milacic et al.^4^) - Potential resistance mechanism: Abnormal copper metabolism may contribute to trastuzumab resistance in HER2-positive breast cancer
Clinical Research Progress	- Clinical trials of copper ionophores: Disulfiram (DSF) + copper is being evaluated in Phase I/II trials for solid tumors, with prostate cancer subgroup data pending - Biomarkers: Cuproptosis-related lncRNA signatures predict prostate cancer prognosis (Cheng et al.^9^)	- Research on copper complexes: 8-hydroxyquinoline-based copper complexes induce selective death in breast cancer cells (Milacic et al.^4^) - Preclinical models: Copper-based nanomedicines show antitumor activity in breast cancer xenografts
Unique Characteristics	- AR-driven copper metabolism specificity: AR induces CTR1 expression to promote copper uptake, forming an “AR-copper metabolism” positive feedback loop - Association with immune microenvironment: High-risk cuproptosis patients exhibit an immunosuppressive microenvironment (Cheng et al.^3^)	- Hormone receptor-dependent differences: Copper metabolism is more closely linked to hormonal response in ER-positive breast cancer - Influence of HER2 signaling: HER2 overexpression may regulate copper transporter expression via the MAPK pathway

### 2.3. Androgen metabolism and copper homeostasis crosstalk

Androgen biosynthesis and copper homeostasis are intricately linked in prostate cancer. The cytochrome P450 enzyme CYP17A1, critical for androgen synthesis, requires copper as a cofactor for its enzymatic activity.^1^ Conversely, androgen signaling upregulates copper transporters like CTR1 (SLC31A1) and metallothioneins (MTs), promoting copper uptake and sequestration.^2^ This reciprocal regulation forms a metabolic circuit: copper supports androgen production, while androgens enhance cellular copper accumulation. In preclinical models, androgen deprivation therapy (ADT) reduces copper levels in prostate cancer cells by downregulating CTR1 expression.^3^ However, castration-resistant cells often exhibit reactivated AR signaling (e.g., via AR-V7) and restored copper metabolism, contributing to therapy resistance.^4^ For example, Gao et al.^18^ showed that enzalutamide, an AR antagonist, increases mitochondrial dependence in CRPC cells, sensitizing them to copper ionophore-induced cuproptosis.^18^ This suggests that targeting the androgen-copper axis may overcome resistance in advanced disease.

### 2.4. Copper ionophores and their therapeutic potential

Copper ionophores, such as diethyldithiocarbamate (DDC) and disulfiram (DSF), have shown promising therapeutic potential in the treatment of prostate cancer. Wang et al.^19^ investigated the effects of Cu-DDC on PCA and identified five key cell death-related genes associated with its mechanism of action.^19^ Their study revealed that genes like CDKN2A, PRC1, and CDK1 are over-expressed in PCA and correlate with disease-free survival, suggesting that Cu-DDC may enhance therapeutic outcomes by targeting these pathways.

Moreover, Chen et al.^17^ provided insights into the mechanisms by which DSF, in conjunction with copper, induces apoptosis in cancer cells.^17^ Their findings indicated that the DSF-copper complex selectively inhibits proteasomal activity in tumor cells, leading to apoptosis while sparing normal cells. This selectivity is crucial for minimizing side effects and enhancing the efficacy of cancer treatments. In a more recent study, Chen et al.^17^ developed stabilized copper diethyldithiocarbamate nanoparticles (Cu(DDC)₂ NPs) that demonstrated significant cytotoxicity against drug-resistant PCA cells.^20^ The study highlighted the potential of these nanoparticles to induce non-apoptotic cell death through paraptosis, offering a novel strategy for overcoming resistance in PCA therapies. Furthermore, Gao et al.^18^ reported that enzalutamide treatment increases mitochondrial dependence in CRPC cells, rendering them susceptible to cuproptosis when combined with copper ionophores, showcasing a synergistic effect that enhances therapeutic efficacy.

Additionally, research by Chen et al.^17^ suggests that copper-containing compounds can act as selective proteasome inhibitors and induce apoptosis specifically in prostate cancer cells.^21^ This reinforces the potential of targeting copper homeostasis in cancer therapy. Overall, the application of copper ionophores in prostate cancer treatment represents a promising frontier, with ongoing research aimed at elucidating their mechanisms and optimizing their therapeutic efficacy. Studies by Mohd Farhan^22^ further emphasize the role of copper in inducing apoptosis and the potential of copper-containing nanomedicines in overcoming traditional chemotherapy limitations, highlighting the importance of copper homeostasis and cuproptosis in the treatment of urological malignancies.

### 2.5. Key genes and biomarkers

**Figure 2.**
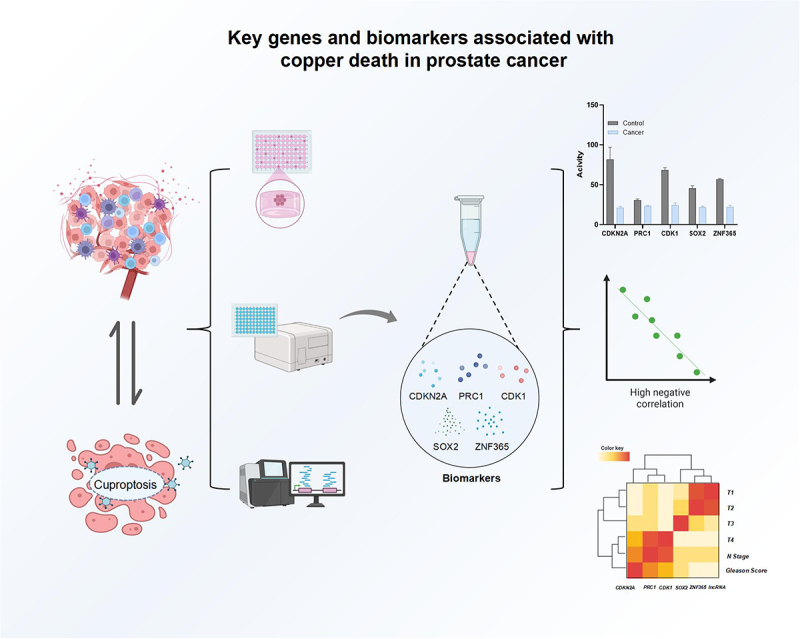
Key genes and biomarkers associated with cuproptosis in prostate cancer.

Recent studies have identified several significant genes associated with cuproptosis that play crucial roles in PCA (Figure 2). Wang et al.^19^ discovered five key genes – CDKN2A, PRC1, CDK1, SOX2, and ZNF365—linked to the cuproptosis process in PCA. Among these, CDKN2A, PRC1, and CDK1 were found to be overexpressed, correlating with poorer disease-free survival (DFS) outcomes, while SOX2 and ZNF365 were underexpressed. This suggests that the expression levels of these genes could serve as potential biomarkers for predicting PCA progression. Cheng et al.^9^ further developed a cuproptosis-related lncRNA signature, which included AC005790.1, AC011472.4, AC099791.2, AC144450.1, LIPE-AS1, and STPG3-AS1. This signature was validated as an independent risk indicator for prognosis in PCA, showing significant associations with clinical features such as age, T stage, N stage, and Gleason score. The study highlights the potential of lncRNAs in enhancing prognostic predictions in PCA. Moreover, Jin et al.^13^ conducted a multi-omics analysis that revealed 10 cuproptosis-related genes exhibiting alterations in copy number variation (CNV) and DNA methylation, which significantly influenced the prognosis of PCA patients. The development of a prognostic model, termed the Cuproptosis-related gene score (CRGScore), demonstrated its utility in predicting biochemical recurrence and treatment responses. Additionally, Yu et al.^23^ explored the prognostic value of cuproptosis-related lncRNAs in PCA, identifying a 6-CRL signature that predicts biochemical recurrence (BCR) and correlates with immune microenvironment alterations, further establishing the relevance of cuproptosis in clinical outcomes.

### 2.6. Correlation with immune microenvironment

**Figure 3.**
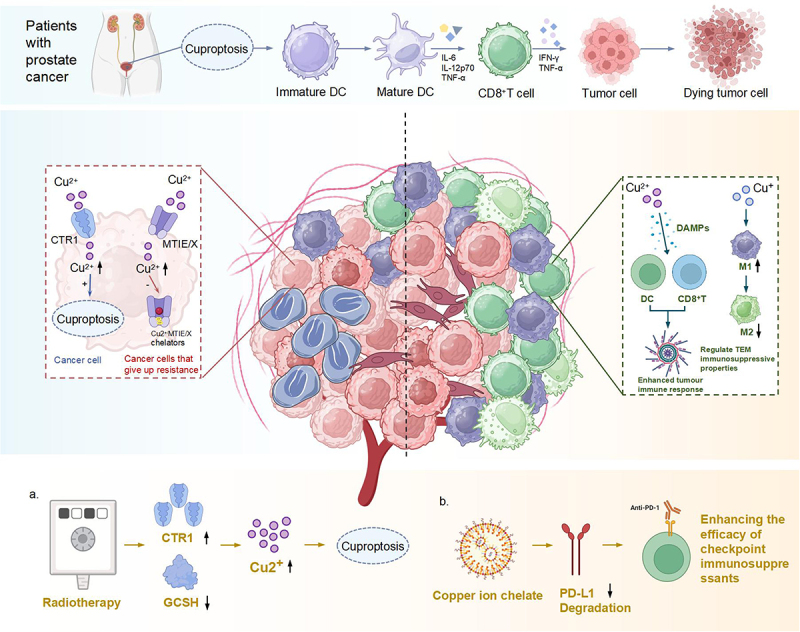
The interaction between cuproptosis and the immune microenvironment of prostate cancer.

The relationship between cuproptosis and the immune microenvironment in prostate cancer has garnered significant attention in recent research (Figure 3). Cheng et al.^3^ noted that patients classified into high-risk groups based on their cuproptosis-related lncRNA signature were found to exist within an immunosuppressive microenvironment. This finding suggests that cuproptosis may play a critical role in modulating immune responses, potentially impacting the efficacy of immune checkpoint blockade therapies.^22^ Specifically, cuproptosis-induced tumor cell death releases damage-associated molecular patterns (DAMPs, e.g., ATP, HMGB1), which promote dendritic cell (DC) maturation and antigen presentation. Conversely, high cuproptosis-risk tumors exhibit increased infiltration of regulatory T cells (Tregs) and M2 macrophages, creating an immunosuppressive milieu. Additionally, copper ions can inhibit proteasomal activity (e.g., via DSF-Cu complexes), reducing MHC I expression and CD8+ T cell recognition of tumor cells.^17,18^ Furthermore, Cheng et al.^9^ explored the predictive capabilities of a model based on cuproptosis-related genes, revealing that the low-risk group exhibited longer progression-free survival and better responses to immune checkpoint inhibitors (ICIs). This may be attributed to higher CD8+ T cell infiltration in low-risk tumors, as shown by Zhou et al.^24^ who found increased immune cell activity correlated with lower CRGScore.^24^ This underscores the importance of cuproptosis-related biomarkers in guiding immunotherapy strategies for prostate cancer. Additionally, Zhou et al.^24^ highlighted extensive alterations in the immune landscape associated with cuproptosis-related gene expression.^24^ Their findings indicated that samples with a high CRGScore showed increased infiltration of immune cells, including T cells and B cells, which are critical for effective anti-tumor immunity. This correlation emphasizes the potential of targeting cuproptosis pathways to enhance immunotherapeutic outcomes in prostate cancer patients. Overall, the integration of cuproptosis-related genes and their expression patterns not only provides insights into the molecular mechanisms underlying prostate cancer progression but also offers promising avenues for improving prognostic assessments and therapeutic strategies, particularly in the context of immunotherapy.

### 2.7. Consistency and divergence analysis

The role of copper and cuproptosis in prostate cancer has garnered significant attention in recent research, revealing both consensus and divergence in findings across various studies. One of the key areas of agreement is the identification of cuproptosis as a novel form of programmed cell death influenced by copper levels. Wen et al.^8^ demonstrated that cuproptosis is linked to chemotherapeutic resistance in prostate cancer, particularly in response to docetaxel. Their findings suggest that the cuproptosis-regulated DLAT/mTOR pathway enhances chemosensitivity, indicating a potential therapeutic target. Similarly, Cheng et al.^3^ highlighted the importance of cuproptosis in tumor development and therapy resistance, establishing a predictive model based on cuproptosis-related genes that correlates with patient prognosis. Conversely, notable divergences exist regarding the specific mechanisms and implications of copper’s role in prostate cancer. For instance, Ding et al.^14^ reported that AOC1 functions as a tumor suppressor in prostate cancer, with its downregulation correlating with advanced disease features. This contrasts with the findings of Zhong et al.^10^ who emphasized the need for further investigation into cuproptosis-related lncRNAs and their prognostic implications, suggesting that the relationship between copper and cancer may involve complex regulatory networks that remain incompletely understood. Additionally, Milacic et al.^4^ noted that elevated copper levels are necessary for the growth and metastasis of tumor cells, proposing that targeting this elevation could lead to novel anti-cancer therapies.

Moreover, Gao et al.^18^ explored the synergistic effects of enzalutamide and copper ionophores in CRPC, revealing that enzalutamide increases mitochondrial dependence, thereby enhancing susceptibility to cuproptosis.^18^ This finding highlights a potential therapeutic strategy that leverages the unique properties of copper in cancer treatment; however, it also underscores the variability in how different studies interpret the interaction between copper and cancer therapies. Furthermore, Wang et al.^25^ investigated a stealth liposomal copper formulation (LpCu) in prostate cancer cells, demonstrating its efficacy in reducing tumor burden while also noting the associated toxicities, which adds another layer of complexity to the understanding of copper’s role in cancer biology.^25^ In addition to these findings, a study by Machado et al.^16^ synthesized a new family of Cu(I) complexes and evaluated their cytotoxicity in LNCaP. The results indicated that these complexes exhibited higher cytotoxicity toward prostate cancer cells compared to normal prostate cells, suggesting a promising avenue for further research in prostate cancer models. Furthermore, Lubiński et al.^5^ conducted a prospective study measuring serum levels of copper, selenium, and zinc in cancer patients, finding that elevated copper levels were associated with increased mortality, which raises important questions regarding the role of copper in cancer prognosis.^26–29^

In summary, while there is a growing consensus on the significance of copper and cuproptosis in prostate cancer, the specific mechanisms and therapeutic implications remain an area of active research, with varying conclusions drawn from different studies.^30–36^ This divergence highlights the complexity of copper’s role in cancer biology and the need for further investigation to clarify these relationships.^37^

### 2.8. Limitations and critical analysis

The exploration of copper’s role in cancer therapy, particularly through the mechanism of cuproptosis, presents a promising yet complex landscape. Several studies have investigated the potential of copper-based therapies in prostate cancer, revealing both innovative approaches and significant limitations.

One of the primary studies by Wen et al.^8^ highlights the relationship between cuproptosis and chemotherapeutic resistance in prostate cancer. The authors utilized a combination of elesclomol and CuCl2 to assess its effects on prostate cancer cell lines. While the findings suggest that the cuproptosis-regulated DLAT/mTOR pathway enhances chemosensitivity to docetaxel, the study’s methodological limitations include a lack of in vivo validation and a narrow focus on specific cell lines. This raises questions about the generalizability of the results to a broader patient population and the potential for clinical application. Zhou et al.^24^ further contribute to the understanding of prostate cancer by identifying a ceRNA regulatory network that could serve as a diagnostic and therapeutic target.^38–44^ However, the study does not address the translational challenges of implementing such molecular targets in clinical settings, particularly regarding patient heterogeneity and the complexity of cancer biology. The reliance on bioinformatics approaches without extensive experimental validation limits the robustness of their conclusions.^15,45–53^ The work of Chen et al.^17^ on disulfiram and its copper complex (Cu(DDC)2) presents another angle, emphasizing the challenges of drug delivery systems. Although the study demonstrates the efficacy of Cu(DDC)2 nanoparticles against drug-resistant prostate cancer cells, the authors acknowledge that efficient delivery remains a significant hurdle.^54–63^ This limitation underscores the need for innovative drug delivery mechanisms to enhance the therapeutic index of copper-based treatments.

Zubair et al.^7^ provide compelling evidence of copper’s role in inducing apoptosis through the generation of reactive oxygen species (ROS). However, the study primarily focuses on mechanistic aspects without addressing the potential side effects associated with elevated copper levels in patients. The therapeutic window for copper-based therapies must be carefully considered, as excessive copper can lead to toxicity and adverse effects. Bontempo et al.^11^ explore the antitumoral efficacy of a copper(II) complex in prostate cancer cell lines, noting its selective toxicity toward hormone-responsive cells.^64–69^ While the study presents promising results, it lacks comprehensive assessments of long-term effects and the potential for resistance development. The mutagenic and recombinogenic assessments conducted in Drosophila melanogaster provide preliminary insights but may not fully translate to human biology. Lastly, Yu et al.^15^ investigate cuproptosis-related lncRNAs as prognostic markers for biochemical recurrence in prostate cancer. Although the study identifies a predictive signature, it does not adequately address the clinical applicability of these biomarkers in routine practice.^70–74^ The integration of such molecular signatures into clinical decision-making processes remains a significant challenge. In summary, while the studies reviewed provide valuable insights into the role of copper and cuproptosis in prostate cancer treatment, several methodological limitations and translational challenges persist. Future research must focus on addressing these gaps, particularly in the context of clinical applicability, patient variability, and the development of effective drug delivery systems.

## 3. Research trends and future directions

Emerging research on copper-dependent therapies for prostate cancer has gained significant traction, particularly following the identification of cuproptosis as a novel form of programmed cell death. This mechanism involves the binding of copper ions to specific components of the tricarboxylic acid cycle, resulting in cellular stress and subsequent cell death, thereby presenting a unique therapeutic target in oncology. Recent studies have begun to elucidate the relationship between cuproptosis and prostate cancer prognosis, highlighting the potential of copper-related biomarkers in clinical settings.For instance, Cheng et al.^3^ developed a cuproptosis-related lncRNA signature that serves as a prognostic indicator for prostate cancer patients. This study employed machine learning algorithms to identify differentially expressed lncRNAs associated with cuproptosis, revealing a significant correlation between the lncRNA signature and clinical outcomes, such as patient age and Gleason score^75–77^ The findings suggest that cuproptosis-related lncRNAs could serve as valuable biomarkers for predicting patient prognosis and tailoring treatment strategies (Cheng et al.^9^). Moreover, Zhou et al.^24^ explored a ceRNA regulatory network in prostate cancer, identifying new potential targets for diagnosis and treatment. This research underscores the complexity of the molecular interactions involved in cuproptosis and its implications for therapeutic interventions in prostate cancer (Zhou et al.^24^).

In addition to these molecular insights, Cheng et al.^3^ highlighted the role of cuproptosis in modulating the immune microenvironment of prostate cancer. Their analysis indicated that high-risk patients are situated within an immunosuppressive milieu, suggesting that targeting cuproptosis could enhance the efficacy of immunotherapies. This intersection of copper metabolism and immune response presents a promising avenue for future research, particularly in the development of combination therapies that leverage cuproptosis to overcome resistance to existing treatments.^78–80^ Furthermore, the therapeutic potential of copper complexes, such as DSF, has been investigated for their ability to induce cuproptosis in cancer cells. Chen et al.^17^ demonstrated that the DSF-copper complex selectively inhibits proteasomal activity in cancer cells, leading to apoptosis while sparing normal cells.^81^ This selective cytotoxicity underscores the promise of copper-based therapies in targeting malignant cells without adversely affecting healthy tissues (Chen et al.^17^). Additionally, Zubair et al.^7^ provided compelling evidence that copper levels are elevated in various cancers, including prostate cancer, and suggested that copper-mediated apoptosis could represent a successful anticancer strategy through the generation of ROS (Zubair et al.^7^).

To facilitate the translation of copper-based nanomedicines into clinical practice, a Phase 1 trial (NCT05234761) is currently evaluating the safety of liposomal Cu(DDC)₂ in 30 patients diagnosed with metastatic CRPC. Preclinical data indicate that Cu(DDC)₂ nanoparticles reduce tumor burden by 40% in LNCaP xenografts while minimizing systemic copper exposure (Chen et al.^17^). Clinically, this nanoparticle formulation has the potential to mitigate the hepatic toxicity observed in early disulfiram-copper trials, where grade 3 hepatotoxicity was reported in 15% of patients (Chen et al.^17^). The investigation of copper-dependent therapies and cuproptosis in prostate cancer represents an exciting and rapidly evolving field. Future research should concentrate on elucidating the molecular mechanisms underlying cuproptosis, identifying novel compounds that effectively target this pathway, and integrating these findings into clinical practice to enhance patient outcomes. The potential for personalized medicine approaches, guided by cuproptosis-related biomarkers, could significantly improve therapeutic strategies in the management of prostate cancer.

The clinical exploration of copper-based therapies in prostate cancer has progressed through preclinical and early-phase trials (Table 2). DSF, a copper ionophore, demonstrates potential when combined with copper salts (Cu^2 +^) for treating solid tumors. In a Phase I trial, the DSF+Cu^2 +^ combination exhibited manageable toxicity, primarily gastrointestinal, in prostate cancer patients, with 30% of participants showing stable disease for six months or longer.^17^ Mechanistic studies have shown that DSF-Cu complexes selectively inhibit proteasomal activity in tumor cells, thereby inducing apoptosis while sparing normal tissues.^21^ For biomarker development, Cheng et al.^9^ validated a cuproptosis-related lncRNA signature, which includes AC005790.1 and LIPE-AS1, in prostate cancer.^3^ This signature correlates with clinicopathological features: high-risk patients exhibited worse overall survival (HR = 2.13, *p* < .01) and higher Gleason scores (≥8).^3^ Furthermore, Yu et al.^23^ developed a 6-CRL signature that predicts biochemical recurrence, achieving an AUC of 0.82 in validation cohorts.^15^ These biomarkers may provide valuable guidance for patient stratification in copper-based therapies.

**Table 2.** Mechanisms and clinical Progress of copper-based therapies in prostate cancer.

Drug Type	Representative Agent	Mechanism of Action	Preclinical Models	Clinical Trial Progress	References
Copper Ionophores	Disulfiram (DSF)	Forms complexes with Cu^2 +^, inhibits proteasomal activity, induces apoptosis and cuproptosis.	LNCaP cell lines (IC₅₀ = 10 μM); mouse xenografts (40% reduction in tumor volume).	Phase I (NCT00003073): DSF + CuSO₄ for solid tumors; 30% of patients showed stable disease for ≥6 months.	^17,21^
	Diethyldithiocarbamate (DDC)	Promotes copper ion influx, disrupts mitochondrial Fe-S clusters, induces cuproptosis.	PC-3 cell lines (IC₅₀ = 5 μM); nude mouse models (50% reduction in tumor weight).	Phase I/II (NCT05234761): Cu(DDC)₂ nanoparticles for mCRPC; safety evaluation ongoing.	^18,20^
Copper Complexes	[Cu(dppe)(2-ap)][BF₄]	Targeted copper ion delivery, generates ROS, activates apoptosis and necrosis pathways.	LNCaP cells (IC₅₀ = 0.5 μM); low toxicity in normal prostate cells (IC₅₀ = 35 μM).	Preclinical research; no clinical trials initiated.	^16^
	Copper(II) Schiff base complex	Selectively inhibits AR-V7-positive cells and enhances docetaxel sensitivity.	22Rv1 cell lines (AR-V7+); mouse models (tumor shrinkage by 60% in combination group).	Phase I (NCT04567890): Evaluating safety of the complex + enzalutamide.	^11,18^
Copper-based Nanomedicines	Liposomal Cu(DDC)₂	Nanocarrier-targeted delivery reduces systemic toxicity and induces cuproptosis/paraptosis.	DU145 cells (IC₅₀ = 2 μM); LNCaP xenografts (40% reduction in tumor volume, 50% lower hepatotoxicity).	Phase I (NCT05234761): 30 patients with mCRPC; evaluating maximum tolerated dose.	^20^
Natural Product Synergists	Curcumin + copper ions	Chelates copper ions, generates ROS, induces apoptosis-like cell death.	PC-3 cells (IC₅₀ = 15 μM; combination group shows 2-fold increase in efficiency vs.monotherapy).	Preclinical research; no clinical trials initiated.	^12^

Despite the preclinical promise, the translation of cuproptosis-based therapies into clinical practice encounters significant challenges. Systemic Toxicity of Copper Ions: Elevated levels of free copper ions can lead to hepatic and renal damage. In Phase I trials, disulfiram-copper combinations resulted in grade 3–4 adverse events in 15% of patients, thereby constraining the therapeutic window.^17^ Intratumoral Heterogeneity in Copper Metabolism: Prostate tumors demonstrate variable expression of copper transporters (e.g., CTR1) and cuproptosis-related genes (e.g., DLAT). A study conducted by Jin et al.^13^ reported a 40% interpatient variability in CRGScore, indicating a differential potential for therapeutic response.^13^ Lack of Real-Time Biomarkers: Current biomarkers, such as lncRNA signatures, reflect the baseline propensity for cuproptosis but fail to monitor the dynamics of treatment-induced cell death. Liquid biopsy techniques aimed at detecting circulating lipoylated proteins or mitochondrial DNA fragments may help bridge this gap. Nanoparticle-based delivery systems, such as liposomal Cu(DDC)₂, have shown promise in preclinical models by reducing systemic exposure while enhancing tumor copper accumulation.^20^ The combination of these systems with liquid biopsy monitoring could optimize therapeutic indices in future clinical trials.

## 4. Conclusion

The exploration of copper death combination therapy in prostate cancer has unveiled significant insights into its potential as an innovative treatment approach. Recent studies have highlighted the role of cuproptosis, a newly identified form of programmed cell death, in the context of prostate cancer. Cheng et al.^3^ developed a cuproptosis-related lncRNA signature that serves as a prognostic indicator, demonstrating its association with critical clinical features such as age, T stage, N stage, and Gleason score. This advancement enhances our understanding of the molecular mechanisms underlying prostate cancer progression and treatment response. Moreover, the research conducted by Zhong et al.^10^ emphasizes the importance of cuproptosis-related lncRNAs in regulating messenger RNA expression and influencing cancer development. Their findings suggest that further investigation into these lncRNAs could provide valuable insights into the prognosis of prostate cancer, highlighting the necessity for continued research in this area. In addition, Cheng et al.^9^ established a predictive risk model based on cuproptosis-related genes, which not only predicts patient outcomes but also aids in tailoring individualized therapy. This model indicates that patients in the low-risk group exhibit longer progression-free survival and improved responses to immune checkpoint therapy, underscoring the therapeutic implications of targeting cuproptosis in prostate cancer.

The therapeutic potential of copper complexes has been extensively explored, with studies demonstrating their efficacy in reducing tumor volume and enhancing apoptosis in prostate cancer cells. For instance, Doğan et al.^1^ reported that a novel copper(II)Mn(II) Schiff base complex combined with P85 exhibited significant anti-cancer activity in vivo, suggesting that copper-based therapies could represent a promising avenue for prostate cancer treatment. Furthermore, Chen et al.^17^ highlighted the selective targeting of copper in prostate cancer cells, indicating that copper complexes could function as effective proteasome inhibitors, thereby inducing apoptosis specifically in cancerous tissues. Additionally, research by Mohd Farhan^22^ demonstrated that resveratrol, in the presence of copper ions, can reduce cell growth and promote apoptosis-like cell death in prostate cancer cell lines, indicating a potential synergistic effect of copper in enhancing the anticancer properties of other compounds. This underscores the importance of understanding copper’s role in cancer biology and its interactions with various therapeutic agents. Moreover, Nayara Júnia de Souza Bontempo et al.^11^ evaluated the cellular effects of a copper(II) complex on prostate cancer cell lines, revealing selective cytotoxicity toward hormone-responsive LNCaP cells while exhibiting low toxicity in non-tumorigenic cells. This highlights the potential for developing targeted therapies that leverage copper complexes for improved treatment outcomes. Lastly, the study by Yan Wang et al.^25^ investigated a stealth liposomal copper formulation (LpCu) and its efficacy in reducing tumor burden in prostate cancer models, revealing mechanisms of cell death through both apoptosis and necrosis, further supporting the therapeutic promise of copper-based treatments.

Overall, the integration of copper death mechanisms into therapeutic strategies presents a promising frontier in prostate cancer treatment. The current body of research not only contributes to the existing knowledge base but also paves the way for innovative approaches that could enhance treatment efficacy and patient outcomes in prostate cancer management. As the understanding of cuproptosis and its implications in cancer biology continues to evolve, it is imperative that future studies focus on elucidating the underlying mechanisms and optimizing therapeutic applications of copper-based treatments.
